# Nonlinear multiplicative dendritic integration in neuron and network models

**DOI:** 10.3389/fncom.2013.00056

**Published:** 2013-05-08

**Authors:** Danke Zhang, Yuanqing Li, Malte J. Rasch, Si Wu

**Affiliations:** ^1^School of Automation Science and Engineering, South China University of TechnologyGuangzhou, China; ^2^State Key Lab of Cognitive Neuroscience and Learning, Beijing Normal UniversityBeijing, China

**Keywords:** shunting inhibition, persistent activity, spiking network model, dendritic computation, mean-field analysis

## Abstract

Neurons receive inputs from thousands of synapses distributed across dendritic trees of complex morphology. It is known that dendritic integration of excitatory and inhibitory synapses can be highly non-linear in reality and can heavily depend on the exact location and spatial arrangement of inhibitory and excitatory synapses on the dendrite. Despite this known fact, most neuron models used in artificial neural networks today still only describe the voltage potential of a single somatic compartment and assume a simple linear summation of all individual synaptic inputs. We here suggest a new biophysical motivated derivation of a single compartment model that integrates the non-linear effects of shunting inhibition, where an inhibitory input on the route of an excitatory input to the soma cancels or “shunts” the excitatory potential. In particular, our integration of non-linear dendritic processing into the neuron model follows a simple multiplicative rule, suggested recently by experiments, and allows for strict mathematical treatment of network effects. Using our new formulation, we further devised a spiking network model where inhibitory neurons act as global shunting gates, and show that the network exhibits persistent activity in a low firing regime.

## 1. Introduction

A hallmark of neural computations is the interaction of excitatory and inhibitory drives (Wilson and Cowan, 1972; Brunel, 2000; Herz et al., 2006; Vogels and Abbott, 2009). Dendritic integration of excitatory and inhibitory synaptic potentials is a complicated biophysical process and involves many non-linearities (Mainen and Sejnowski, 1996; Borg-Graham et al., 1998; London and Häusser, 2005). In particular, the position of the presynaptic inputs to a particular neuron on the dendritic tree is very important for signal integration and potential spike initiation (Cristo and Ango, 2007). For instance, if an excitatory input occurred in the distal region of the dendritic tree (far away from the soma) it is possible that a simultaneous inhibitory input to the proximal region (near to the soma) could effectively “shunt” the excitatory pulse on its way to the soma. Accordingly, this effect is called shunting inhibition [Barrett and Crill, 1974; Blomfield, 1974; Rall, 2011, see also review in Koch (2004)]. On the other hand, if the position of the two synapses on the dendritic tree would be exchanged, a simultaneous input would have a markedly different outcome, with the excitatory pulse reaching the soma and potentially causing the generation of a spike.

While the theoretical basis for the effect of shunting inhibition has been laid out several decades ago by analyzing passive electric membrane properties of dendrites (Barrett and Crill, 1974; Blomfield, 1974; Rall, 2011; Koch, 2004), neural network models today still often rely on single compartment models (point models), such as (quadratic) integrate-and-fire model (Gerstner and Kistler, 2002; Izhikevich, 2006), and thus ignore this potentially important non-linear integration of synaptic inputs (McLaughlin et al., 2000; Wang, 2002; Vogels and Abbott, 2009; Rasch et al., 2011).

Acknowledging this inaccuracy of common network models already decades ago, it was shown that incorporating non-linear dendritic effects, such as shunting inhibition, into the mean-field equations of a network of neurons is indeed possible (Abbott, 1991a,b). These non-linear effects are potential important as they e.g., explain persistent activity of low firing rates which cannot be well explained by common models as they typically fire in the saturation regime of the input–output relation with unreasonably high firing rates (>100 Hz; Gold and Shadlen, 2001). However, by starting from the full cable equation, the equations in Abbott (1991b) for incorporating the non-linear dendritic effects are not of a simple mathematical form.

Indeed, it is known that the effect of shunting inhibition can be (at least during steady state) mathematically conceptualized simply as a “dirty multiplication” of excitatory and inhibitory input conductances (Koch et al., 1982; Koch, 2004) in contrast to the common form of linear summation of any two inputs in simple neuron models. Accordingly, a recent study, re-investigating shunting inhibition experimentally (Hao et al., 2009), found that indeed the integration of simultaneous excitatory post-synaptic potentials (EPSP) and inhibitory post-synaptic potentials (IPSP) could be well described in a multiplicative form:

(1) $$\Delta V_{\text{Soma}}\propto \text{EPSP}+\text{IPSP}+\kappa \cdot \text{EPSP}\cdot \text{IPSP},$$

where κ is a factor determining the strength of the shunting effect that depends on the spatial arrangement of the synaptic inputs on the dendritic tree.

Given the good experimental agreement of this description, a follow-up study tried to incorporate this abstract formulation of shunting inhibition into a simple single-compartment neuron model (Zhou et al., 2013), which the authors called the “DIF”-model (dendritic integrate-and-fire). In fact, incorporating non-linear effects of shunting inhibition into simple neuron models would make neural network simulations based on point models more realistic while keeping the formulation and simulation simple and efficient: there would be no need to simulate complex multi-compartment neuron models instead where effects of shunting inhibition are very well understood (Hao et al., 2009).

However, in the study (Zhou et al., 2013), the derivation was based on phenomenological arguments to describe the effects of shunting as observed in experiments. Here we suggest a different derivation of a single compartment model starting from a biophysical more realistic conductance-based three-compartment model. The advantage of our derivation is that the shunting strength κ can be approximated analytically in biophysical terms. Moreover, our formulation naturally includes the correct distance dependence of the shunting strength κ and the experimental results of Hao et al. (2009) can be captured well.

The usefulness of integrating non-linear dendritic processing into a simple neuron model is that network effects induced by shunting inhibition can now be analyzed in much simpler mathematical form. For that, we further extend our model to include multiple excitatory and inhibitory sites with specific layout on the dendritic branches and devised a network of mutually connected excitatory and inhibitory groups of neurons. The structure of our network model is similar to those analyzed earlier (Wilson and Cowan, 1972; Brunel, 2000), and derivations of its topology were suggested for many brain areas, such as for memory circuits (Hopfield, 1984), pre-frontal cortex computations (Machens et al., 2005), or decision making (Wang, 2002; Wong and Wang, 2006). In contrast to previous models, however, in our network inhibitory neurons act as global shunting gates, and thus introduce a new multiplicative non-linearity. In fact, this arrangement of inhibitory inputs—specifically targeting the peri-somatic regions to act as global shunting gates—has been found to be a common motif for certain basket cell types in the brain (Markram et al., 2004).

We show that our simplified mathematical form of non-linear dendritic inputs is capable of generating persistent activity with relatively low firing rate in simulations of networks of spiking neuron (around 15 Hz), as has previously been found for firing rate models using a more detailed incorporation of dendritic effects (Abbott, 1991a). Moreover, firing levels can be “adjusted” in a gradual manner by e.g., changing the excitatory drive.

Non-linear dendritic processing might be an important underlying mechanism for commonly observed spiking dynamics in the brain. Our work suggests that non-linear dendritic effects can be simply incorporated in networks of spiking neurons.

Parts of the results were previously presented on a conference (Zhang et al., 2011).

## 2. Results

To get an intuitive idea of the non-linearity involved in integrating two synaptic inputs, let us first consider a conductance-based neuron model which receives a pair of excitatory and inhibitory inputs at the soma. The dynamics of the neuron can be written as Koch (2004)

(2) $$C\frac{dv}{dt}=-g(v-E_{L})-g^{E}(v-E_{E})-g^{I}(v-E_{I}),$$

where *v* is the membrane potential of the neuron and *C* the membrane capacitance. *g* is the leaky conductance and *E*_L_ the resting potential. *g*^E^ and *g*^I^ denote, respectively, the excitatory and inhibitory conductances, and *E*_E_ and *E*_I_ the corresponding reversal potentials. One finds from Equation (2) that the membrane potential in the steady state can be written as

(3) $$\bar{v}=E_{L}+\frac{g^{E}}{\gamma }(E_{E}-E_{L})+\frac{g^{I}}{\gamma }(E_{I}-E_{L}),$$

with the factor γ := *g* + *g*^E^ + *g*^I^. Thus the excitatory conductance *g*^E^, as well as the inhibitory conductance *g*^I^ is scaled by a factor (γ) involving both, the inhibitory and excitatory conductances. Therefore the integration of inhibitory and excitatory currents on the somatic potential is non-linear rather than an independent linear summation.

Of course, shunting interaction in reality is more complicated than this simple case because its effect depends on the spatial configuration of excitatory and inhibitory inputs on the dendrite of a neuron. In the following, we will derive a simple single compartment neuron model which incorporates the effects of shunting inhibition via a multiplicative rule. After the derivation, we will show how different geometric arrangements can be modeled in a network and finally show how shunting inhibition can lead to persistent activity in a neural network.

### 2.1. Derivation of a single compartment model with non-linear dendritic integration

#### 2.1.1. On-path configuration

**Figure 1:**
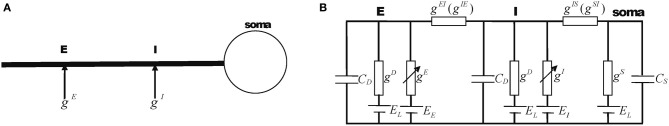
**The three-compartment model. (A)** The spatial configuration of synaptic inputs on the dendrite, with the inhibitory input being on the path from the excitatory site to the soma. **(B)** The equivalent electrical circuit describing the sub-threshold dynamics of the neuron (Equations 4–6).

Let us consider a simple integrate-and-fire neuron model, which consists of a soma and two dendritic compartments. The neuron receives an excitatory and an inhibitory input at the locations E and I on the dendrite, respectively (see Figure 1).

Let us assume that an inhibitory input is on the route from an excitatory input on its way to the soma, i.e., the position I is between E and the soma (Figure 1A). This situation was called the “on-path configuration” (Koch et al., 1983). The sub-threshold dynamics of this neuron can be written (compare to the equivalent circuit in Figure 1B):

(4) $$C_{S}\frac{dv^{S}}{dt}=-g^{S}(v^{S}-E_{L})-g^{IS}(v^{S}-v^{I}),$$

(5) $$C_{\text{D}}\frac{dv^{I}}{dt}=-g^{D}(v^{I}-E_{L})-g^{SI}(v^{I}-v^{S})-g^{EI}(v^{I}-v^{E})-g^{I}(v^{I}-E_{I}),$$

(6) $$C_{\text{D}}\frac{dv^{E}}{dt}=-g^{D}(v^{E}-E_{L})-g^{IE}(v^{E}-v^{I})-g^{E}(v^{E}-E_{E}),$$

where *v*^S^, *v*^I^, and *v*^E^ denote the local membrane potentials at the soma and the dendrite locations I and E, respectively. *C*_S_ and *C*_D_ are the membrane capacitances of the soma and the dendrite locations E and I, respectively. *E*_L_ is the resting potential of the neuron. *E*_I_ and *E*_E_ are the reversal potentials of the inhibitory and excitatory currents, respectively. *g*^S^ and *g*^D^ are, respectively, the leaky conductances at the soma and the dendrite locations. *g*^IS^ is the transfer conductance from the dendritic location I to the soma, and *g*^SI^ the transfer conductance from the soma to the dendritic location I. *g*^IE^ and *g*^EI^ are defined accordingly.

The excitatory and inhibitory synaptic conductances, *g*^E^ and *g*^I^, respectively, are describing the opening of corresponding ion channels. If we assume simple exponential activation curves, the dynamics of the synaptic inputs can be written as Koch (2004)

(7) $$\tau_{E}\frac{dg^{E}}{dt}=-g^{E}+\tau_{E}w_{E}\sum_{m}\delta (t-t^{m})$$

(8) $$\tau_{I}\frac{dg^{I}}{dt}=-g^{I}+\tau_{I}w_{I}\sum_{m}\delta (t-t^{m}).$$

Thus, synaptic conductances are driven by presynaptic spike trains which are expressed as a sum of delta-functions, ∑*_m_δ*(*t* − *t*^*m*^), with *t*^*m*^ denoting the moment of the *m*-th spike. τ_E_ and τ_I_ are the time constants for the excitatory and inhibitory synaptic conductances, respectively. *w*_*E*_ and *w*_*I*_ are the corresponding synaptic connection strengths.

The dynamic of the neuron model of Equations (4–6) is difficult to analyze because it involves the dynamics of three membrane voltages. Thus a further simplification is desirable especially when considering the dynamics of a large network of interacting neurons. In the following, we will further simplify the above model by a separation of time-scales approach.

From Equations (4–6), we note that the magnitudes of the time constants of the potentials at the soma and the dendrite locations I and E can be roughly estimated to be τ_S_ ≈ *C*_S_/(*g*^S^ + *g*^IS^), τ_DI_ ≈ *C*_D_/(*g*^D^ + *g*^SI^ + *g*^EI^) and τ_DE_ ≈ *C*_D_/(*g*^D^ + *g*^IE^). Since the capacitance increases linearly with the surface area of the membrane (see e.g., Koch, 2004), the membrane capacitance at the soma is much larger than that at the dendritic locations E and I, i.e., *C*_S_ » *C*_D_. Furthermore, we assume that the leak conductances *g*^S^ and *g*^D^ are of the same order and larger than the transfer conductances, i.e., *g*^S^» *g*^IS^, *g*^D^» *g*^SI^ and *g*^D^» *g*^IE^. Therefore, we have τ_S_ » τ_DI_ and τ_S_ » τ_DE_. In fact, using the parameters from Table 1 it is τ_DI_ ≈ 1.7 ms, τ_DE_ ≈ 2.3 ms, and τ_S_ ≈ 13.4 ms and therefore indeed τ_S_ » τ_DE_ ≈ τ_DI_. This implies that the dynamics of *v*^I^ and *v*^E^ occur much faster than that of *v*^S^. Thus, we can effectively treat *v*^S^ as a slow time variable, and *v*^I^, *v*^E^ as fast time variables. The dynamics of the somatic potential can then be solved approximately by assuming that *v*^I^ and *v*^E^ reach their steady values instantly. This is achieved by setting the left-hand sides of Equations (5) and (6) to zero and solve for the membrane potentials. We obtain:

(9) $$v^{-I}=E_{L}+\frac{g^{SI}(v^{S}-E_{L})+g^{EI}(v^{-E}-E_{L})+g^{I}(E_{I}-E_{L})}{g^{D}+g^{SI}+g^{EI}+g^{I}},$$

(10) $$v^{-E}=E_{L}+\frac{g^{IE}(v^{-I}-E_{L})+g^{E}(E_{E}-E_{L})}{g^{D}+g^{IE}+g^{E}}.$$

**Table 1:** **Parameters used in the main text and numerical calculations**.

**Description**	**Parameter**	**Values**
Somatic reversal potential	*E*_L_	−70 mV
Exc. reversal potential	*E*_E_	10 mV
Inh. reversal potential	*E*_I_	−80 mV
Somatic membrane capacitance	*C*_S_	740 pF
Dendritic membrane capacitance	*C*_D_	50 pF
Somatic leaky conductance	*g*^S^	30 nS
Dendritic leaky conductance	*g*^D^	20 nS
Transfer conductance soma to I	*g*^SI^	5 nS
Transfer conductance I to soma	*g*^IS^	α *g*^SI^
Transfer conductance soma to E	*g*^SE^	10 nS
Transfer conductance E to soma	*g*^ES^	α *g*^SE^
Transfer conductance I to E	*g*^IE^	1 nS
Transfer conductance E to I	*g*^EI^	α *g*^IE^
Scaling factor	α	5

Further, we assume that the input locations, E and I, and the soma are well separated on the dendritic branch (as this is the experimental condition during which (Hao et al., 2009) obtained their results). Substituting Equations (9) and (10) into Equation (4), we then get a simplified model for the dynamics of the somatic potential (see section 4.1 for a detailed derivation),

(11) $$\begin{array}{l} \tau_{S}\frac{dv^{S}}{dt}=-(v^{S}-E_{L})+f_{\text{d}}(g^{E})+f_{\text{p}}(g^{I})\\ \;+\kappa_{\text{on}}f_{\text{d}}(g^{E})f_{\text{p}}(g^{I}), \end{array}$$

where τ_S_ = *C*_S_/(*g*^S^ + *g*^IS^), and

(12) $$f_{\text{d}}(g^{E})=\frac{g^{IS}g^{EI}g^{E}(E_{E}-E_{L})}{\begin{array}{l} \left(g^{S}g^{D}+g^{S}g^{SI}+g^{S}g^{EI}+g^{D}g^{IS}+g^{EI}g^{IS}\right)\\ \;\times \;(g^{D}+g^{E}+g^{IE}), \end{array}}$$

(13) $$f_{\text{p}}(g^{I})=\frac{g^{IS}g^{I}(E_{I}-E_{L})}{\begin{array}{l} \left(g^{S}g^{D}+g^{S}g^{SI}+g^{D}g^{IS})+g^{I}(g^{S}+g^{IS}\right)\\ \;+\;g^{EI}(g^{S}+g^{IS}), \end{array}}$$

(14) $$\kappa_{\text{on}}=\frac{g^{S}+g^{IS}}{g^{IS}(E_{L}-E_{I})}.$$

To understand the behavior of the new neuron model (Equation 11), it is instructive to look at the steady state of the somatic potential in response to constant synaptic inputs. The steady state ${\bar{v}}^{S}$ is obtained by setting the left-hand side of Equation (11) to be zero:

(15) $${\bar{v}}^{S}=E_{L}+f_{\text{d}}(g^{E})+f_{\text{p}}(g^{I})+\kappa_{\text{on}}f_{\text{d}}(g^{E})f_{\text{p}}(g^{I}).$$

Thus, when no inhibitory input is applied, i.e., *g*^I^ = 0 and *f*_p_(*g*^I^) = 0, then *v*^S^ − *E*_L_ = *f*_d_(*g*^E^) is the voltage change at the soma due to the excitatory input *g*^E^. Analogously, if no excitatory input is applied, i.e., *g*^E^ = 0 and *f*_d_(*g*^E^) = 0, then the voltage change at the soma is given by *v*^S^ − *E*_L_ = *f*_p_(*g*^I^). Thus, in case of single inputs the multiplicative effects of the dendrite is reduced to an additive form as in the common formulation of an integrate-and-fire model. However, if both excitatory and inhibitory inputs are applied simultaneously, their joint effect on the somatic potential is given by *v*^S^ − *E*_L_ = *f*_d_(*g*^E^) + *f*_p_(*g*^I^) + κ_on_*f*_d_(*g*^E^)*f*_p_(*g*^I^), that is a summation of the excitatory and inhibitory contribution when each of them was applied separately, and an additional product of their independent contributions, i.e., κ_on_ *f*_d_(*g*^E^)*f*_p_(*g*^I^). The multiplicative term comes from the non-linear shunting process, and the coefficient κ_on_ represents the shunting strength.

**Figure 2:**
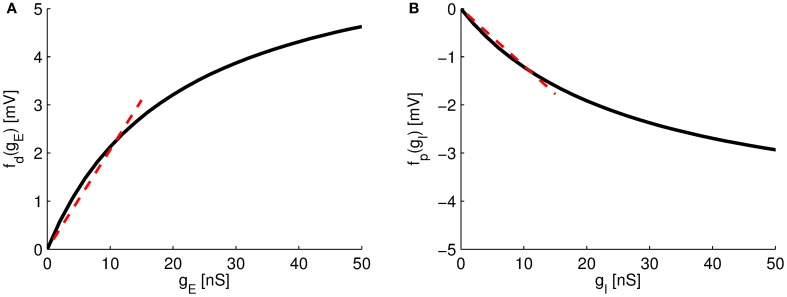
**Somatic response functions. (A)** The function *f*_d_(*g*^E^) of the distal excitatory site. **(B)** The function *f*_p_(*g*^*I*^) of the proximal inhibitory site. For small values of *g*^E^ and *g*^I^, *f*_d_(*g*^E^) and *f*_p_(*g*^I^) can be approximated as linear functions (dotted lines). Parameters as in Table 1.

The new neuron model (Equation 11) describes the sub-threshold voltage dynamics at the soma if two synaptic sites, excitatory and inhibitory synapses, are arranged in on-path configuration. The summation of excitatory and inhibitory conductances agrees with the form found in a recent experiment [Hao et al. (2009); see also Equation (1)], that is a linear sum of individual excitatory or inhibitory effects, *f*_d_(*g*^E^) + *f*_p_(*g*^I^), and a multiplicative term involving both, excitatory and inhibitory currents *f*_d_(*g*^E^)*f*_p_(*g*^I^), as well as a shunting strength factor κ_on_. The functions *f*_d_(*g*^E^), for the *distal* excitatory synaptic site, and *f*_p_(*g*^I^), for the *proximal* inhibitory synaptic site, correspond to the induced voltage change at the soma for a given conductance input at the respective synaptic sites. If input conductance *g*^E^ is small, *g*^E^« *g*^D^, then *f*_d_(*g*^E^) is approximately linear (see Figure 2A). For larger excitatory inputs, the function saturates to a positive value. Analogously, if the inhibitory conductance *g*^I^ is small, *g*^I^« *g*^S^, then the function *f*_p_(*g*^I^) is approximately linear (Figure 2B), but similarly saturates to a negative value for larger inhibitory inputs.

Note that these functions relating the somatic effect of the synaptic conductances include the transfer conductances from one site to the other. They are thus dependent on the distance between the excitatory and inhibitory site, as well as the distance to the soma. If only passive cable properties are considered, transfer conductances in both directions, e.g., *g*^EI^ and *g*^IE^, would be equal (Koch, 2004). In practice, however, depolarization of the membrane potential caused by excitatory currents is amplified by the existence of voltage-dependent ion-channels (Cook and Johnston, 1997; Lai and Jan, 2006). Thus transfer conductances of excitatory input locations to other parts of the dendrite is increased in comparison to the transfer conductances from inhibitory sites. We thus set *g*^EI^ = α*g*^IE^, with α > 1 to reflect this property. Moreover, since in on-path configuration excitatory currents will flow pass the inhibitory location, causing similarly an amplification through active channels, we further set *g*^IS^ = α *g*^SI^. Note that the setting of α does not affect our qualitative results but will influence the size of the somatic voltage change in response to synaptic inputs (see below and Figure 2).

In contrast to the approach of Zhou et al. (2013), in our neuron model (Equation 11) the shunting strength κ_on_ is explicitly given in biophysical terms. From Equation (14) it can be seen that the shunting strength will be particular prominent if the resting potential and the inhibitory reversal potentials are similar *E*_L_ ≈ *E*_I_, in agreement with experimental and early theoretical findings (Koch, 2004; Hao et al., 2009). Note further that κ_on_ decreases with growing transfer conductance *g*^IS^. Thus if location I is set farther away from the soma (while still retaining the on-path configuration), the transfer conductance to the soma *g*^IS^ will naturally decrease, and therefore the shunting will increase. Thus, κ_on_ tends to have a larger value if inhibitory inputs are at a distal site of a dendrite in comparison to inputs at a proximal site, agreeing with experimental observations (Hao et al., 2009). Moreover, in contrast to earlier two-port analysis (Koch et al., 1983; Hao et al., 2009; Zhou et al., 2013), our approximation of κ_on_ does *not* depend on the transfer conductance between E and I, therefore the shunting strength is approximately constant when the excitatory synapse location is increasingly distal but the inhibitory location is fixed. This again agrees well with experimental findings (Hao et al., 2009).

Taken together, we found that in on-path configuration a single somatic point model can be derived starting from a three-compartment model when assuming that dendritic processing is fast compared to the somatic integration and that locations E and I are well separated. In this case the arithmetic rule (Equation 1) as suggested by Hao et al. (2009) is well captured [compare to Equation (15)]. We tested the accurateness of the simplifications by comparing the dependence of κ_on_ on the input conductances. Note that in the simplified model (as in the suggested rule of Hao et al., 2009) κ_on_ is independent of *g*^E^ and *g*^I^. If we instead attempt to compute the shunting strength directly from the full three-compartment model by assuming that the arithmetic rule (Equation 1) was correct, we find that κ_on−full_ is indeed almost not dependent on the input conductances. In detail, we calculated the steady state somatic voltage of the full three-compartment model, and solve for κ according to the arithmetic rule (Equation 1). That is, we first subtract both individual excitatory and inhibitory contributions from the steady state voltage [by setting *g*^I^ = 0 or *g*^E^ = 0, respectively) and then divide the result by both individual contributions (compare to Equation (1)] to get the shunting strength (which might in this case still depend on the input conductances *g*^E^ and *g*^I^).

**Figure 3:**
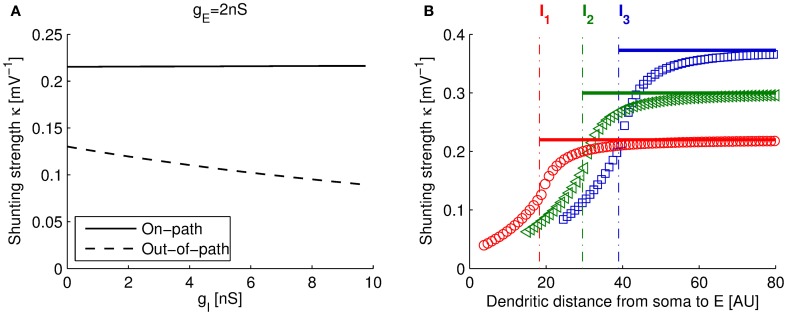
**Dependence of shunting strength with input conductances and distance. (A)** Dependence of the shunting strength with inhibitory input conductance *g*^I^ for the full three-compartment model in on-path and out-of-path configuration (*g*^E^ = 2 nS; dendritic locations (see plot **B**), on-path: *x*(I) = 18, *x*(E) = 50, out-of-path: *x*(I) = 18, *x*(E) = 15). For out-of-path configuration (dashed line), κ is heavily dependent on *g*^I^. In this case, the arithmetic rule (Equation 1) and the single compartment model are less useful. **(B)** Shunting strength κ depends on the spatial arrangement of locations E and I. The plot shows the shunting strength computed with the three-compartment model vs. distance of E from the soma (in arbitrary units), while fixing I at three different locations, I_1_, I_2_, and I_3_ (dotted lines). Solid lines show the dependence of κ_on_ of our single compartment model: κ_on_ is constant (in E) for the on-path configuration (Equation 14).

As plotted in Figure 3A (solid line), the computed κ_on−full_ from the full model (using the parameters of Table 1) changed very little for a large range of *g*^I^ (or *g*^E^, not shown). This indicates that the suggested arithmetic rule of Hao et al. (2009) (Equation 1) is applicable and that our single compartment model is a good approximation to the on-path configuration.

In summary, we derived a new formulation of the sub-threshold dynamics of an integrate-and-fire model with integrated dendritic processing [Equation (11); note that our form of the single compartment model is different from the formulation of Zhou et al. (2013)]. Our model incorporates effects of shunting inhibition of two synaptic inputs with on-path configuration.

#### 2.1.2. Out-of-path configuration

Analogous to the on-path configuration, we can derive a simplified model for the out-of-path configuration, that is, when the excitatory synapse lies on the route of the inhibitory current to the soma. Formally in (Equation 4–6), excitatory and inhibitory synapses exchange position, but the equations otherwise remain the same (that is all labels E and I have to be interchanged in Equation 4–6).

The resulting equations are similar, except that now *f*_d_(*g*^E^) and *f*_p_(*g*^I^) are replaced by *f*_d_(*g*^I^) and *f*_p_(*g*^E^) (with appropriately adapted labeling):

(16) $$f_{\text{p}}(g^{E})=\frac{g^{ES}g^{E}(E_{E}-E_{L})}{\begin{array}{l} \left(g^{S}g^{D}+g^{S}g^{SE}+g^{D}g^{ES})+g^{E}(g^{S}+g^{ES}\right)\\ \;+\;g^{IE}(g^{S}+g^{ES}) \end{array}},$$

(17) $$f_{\text{d}}(g^{I})=\frac{g^{ES}g^{IE}g^{I}(E_{I}-E_{L})}{\begin{array}{l} \left(g^{S}g^{D}+g^{S}g^{SE}+g^{S}g^{IE}+g^{D}g^{ES}+g^{IE}g^{ES}\right)\\ \times \;(g^{D}+g^{I}+g^{EI}) \end{array}},$$

(18) $$\kappa_{\text{out}}=\frac{\left(g^{S}+g^{ES}\right)}{g^{ES}(E_{L}-E_{E})}.$$

To arrive at these equations we used analogous assumptions as in the on-path case. In particular, we assumed that both dendritic locations are well separated. Note, that in out-of-path configuration, the shunting strength is negligible, because the absolute difference of the somatic reversal potential (*E*_L_ ≈ −80 mV) and the excitatory synaptic reversal potential (*E*_E_ ≈ 0 mV) is very large, thus κ_out_ ≈ 0 mV^−1^. This result agrees well with previous theoretical analysis (Koch et al., 1983), where it was found that the shunting strength in out-of-path configuration is negligible compared to that in on-path configuration.

In the derivation of the model for the out-of-path configuration we assumed that all synaptic locations are well separated and found that the arithmetic rule (Equation 1) is approximately satisfied. However, note that our approximation is less successful than in on-path configuration. In particular, if compared to the shunting strength derived from the full model in out-of-path configuration (analogous to the calculation of κ_on−full_ as described above), we find that κ_out−full_ is strongly dependent on the input conductances, specifically on *g*^I^. This dependence is plotted in Figure 3A (dashed line).

Taken together, since both, the arithmetic rule (Equation 1) and our single compartment model (Equation 16), predict a constant shunting strength κ_out_ in respect to the inputs, they are both less valid in out-of-path configuration than in on-path configuration, where κ_on−full_ can indeed be regarded as constant.

#### 2.1.3. Shunting strength depends on the distance between the synaptic sites and the soma

Since we derived our single compartment model (Equation 11) starting from a three-compartment model, distance dependence of the shunting strength can be qualitatively assessed by assuming distance dependence transfer conductances, namely *g*^EI^, *g*^EI^, *g*^IS^, and *g*^SI^. Biophysically, resistance will grow linearly with increasing length of a passive cable (Koch, 2004), thus we assume that each transfer conductance between two points on the dendrite is reversely linearly dependent on their distance *x*.

For estimating the distance dependence of the shunting strength κ, we follow the experiments of Hao et al. (2009) (their Figure 3), and first fix the inhibitory location I at three positions, I_1_, I_2_, and I_3_, and set *g*^SI^ to 2.2, 3, and 5 nS, respectively. The other transfer conductances are given by their distance dependence, *g*_trf_ = *g*_max_/(μ*x* + 1). Here, *g*_max_ is the conductance when two points are co-localized together (*x* = 0), and is much larger than the leaky conductance, we set *g*_max_ = 300 nS. The spatial scaling factor μ depends on the electrotonic properties of dendrite (Koch, 2004) and determines the spatial scale, we set arbitrarily μ = 3 as it does not change the qualitative picture. Other transfer conductances are set according to the spatial arrangement of the input sites I and E, e.g., for the out-of-path configuration it is 1/*g*^*SE*^ + 1/*g*^*IE*^ = 1/*g*^*SI*^. The rest of the parameters are set according to Table 1.

Figure 3B shows the variation of the shunting strength of the full three-compartment model for the on-path configuration (Equations 4–6) and the out-of-path configuration as a function of the distance of the excitatory site from the soma. Note that when varying this distance, the model switches from out-of-path to on-path configuration at the site of the inhibitory input (marked with dashed-dotted lines in Figure 3B). We found that the shunting strength increases sharply for out-of-path configurations the nearer the excitatory and inhibitory sites are, whereas the shunting strength remains approximately constant for the on-path configuration. This distance dependence of our three-compartment model reproduces the experimental findings as well as complex model simulations using dendrites having 200 compartments (Hao et al., 2009). Our single compartment model assumed that inhibitory and excitatory sites are well separated and thus approximates the full model in the asymptotic regime (solid lines in Figure 3B).

### 2.2. Neuron having multiple dendrites

**Figure 4:**
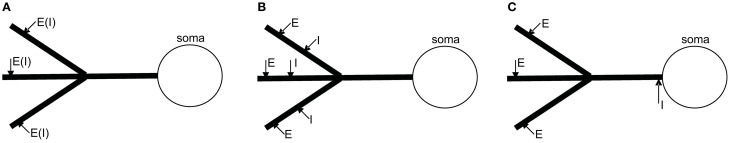
**Three different spatial configurations of multiple synaptic sites on the dendrite. (A)** Individual excitatory and inhibitory inputs are distributed on individual dendritic branches. **(B)** Pairs of excitatory and inhibitory inputs are located on individual dendritic branches. **(C)** Global shunting: the inhibitory input is on the path of all excitatory currents to the soma.

Up to now, we analyzed the shunting contributions to a neuron receiving only two inputs, one excitatory and one inhibitory synapse. However, in real situations as well as in neural network models, a neuron will typically receive hundreds to thousands of input synapses. To investigate the effect of multiple input synapses, we here analyze three possible configurations of synapses on dendritic branches exemplifying potential excitatory and inhibitory interaction patterns (see Figure 4).

#### 2.2.1. Single synapses on individual dendritic branches

In the first configuration, individual excitatory or inhibitory inputs are scattered on different dendritic branches (as illustrates in Figure 4A). In this case, the synapses are physically separated on parallel branches and thus shunting interaction between excitatory and inhibitory currents can be ignored. As shown in the section 4.2.1, the simplified model for the somatic potential *v*^S^ is given by

(19) $$\tau_{S}\frac{dv^{S}}{dt}=-(v^{S}-E_{L})+\sum_{i}f_{N}(g_{i}^{T}),$$

where *g*^T^_*i*_ denotes the corresponding synaptic input conductance of the *i*th synapse and *T* is a reminder of the type of the *i*th synapse, either inhibitory or excitatory. The time constant of the somatic voltage is given by τ_S_= *C*_S_/(*g*^S^ + *Ng*^TS^), where *g*^TS^ denotes the transfer conductance from an input site to the soma. We assume that the transfer conductances from synaptic sites to the soma are equal for all *N* dendritic branches. The function relating the synaptic conductances to the somatic voltage change is given by

(20) $$f_{N}(g_{i}^{T})=\frac{g^{TS}g_{i}^{T}(E_{i}^{T}-E_{L})}{\left(g^{S}+Ng^{TS})(g^{D}+g_{i}^{T}\right)},$$

where *g*^T^_*i*_ is the synaptic conductance on the *i*th dendritic branch and *E*^T^_*i*_ the corresponding reversal potential. Thus, according to Equation (19), if inhibitory and excitatory synapses are located on individual branches, their individual somatic contributions can simply be added.

#### 2.2.2. On-path configuration on each dendritic branch

In the second configuration (Figure 4B), each dendritic branch has a pair of excitatory and inhibitory synapses in on-path configuration. In this case, the simplified model can be written as (see section 4.2.2)

(21) $$\begin{array}{l} \tau_{S}\frac{dv^{S}}{dt}=-(v^{S}-E_{L})\\ \;+\sum_{i}\left[f_{N\text{d}}(g_{i}^{E})+f_{N\text{p}}(g_{i}^{I})+\kappa_{\text{N-on}}f_{N\text{d}}(g_{i}^{E})f_{N\text{p}}(g_{i}^{I})\right], \end{array}$$

where τ_S_ = *C*_S_/(*g*^S^ + *Ng*^IS^), and

(22) $$f_{N\text{d}}(g_{i}^{E})=\frac{g^{IS}g^{EI}g_{i}^{E}(E_{E}-E_{L})}{g^{D}(g^{D}+g_{i}^{E})(g^{S}+Ng^{IS})}$$

(23) $$f_{N\text{p}}(g_{i}^{I})=\frac{g^{IS}g_{i}^{I}(E_{I}-E_{L})}{\left(g^{S}+Ng^{IS})(g^{D}+g_{i}^{I}+g^{IE}\right)}$$

(24) $$\kappa_{\text{N-on}}=\frac{g^{S}+Ng^{IS}}{g^{IS}(E_{L}-E_{I})}.$$

Note that in this configuration, individual shunting components of each branch can be simply added together.

#### 2.2.3. Global shunting

In the third configuration, only excitatory synapses are distributed on dendritic branches and a single inhibitory synapse is located in the peri-somatic region (see Figure 4C). In this configuration, the single inhibitory input might shunt all incoming excitatory currents, and we therefore call it *global shunting*. For simplicity, we assume that the inhibitory synapse is located very close to the soma, so that the inhibitory compartment can be identified with the somatic compartment. This targeting of the perisomatic region can be commonly observed for certain types of basket-cells (Markram et al., 2004). In this case, the following single compartment model can be derived (see section 4.2.3 for details)

(25) $$\begin{array}{l} \tau_{S}\frac{dv^{S}}{dt}=-(v^{S}-E_{L})+\sum_{i}f_{G\text{d}}(g_{i}^{E})+f_{G\text{p}}(g^{I})\\ \;+\;\kappa_{\text{G}}f_{G\text{p}}(g^{I})\sum_{i}f_{G\text{d}}(g_{i}^{E}), \end{array}$$

were τ_S_ ≈ *C*_S_/*g*^S^ and

(26) $$f_{G\text{p}}(g^{I})=\frac{g^{I}(E_{I}-E_{L})}{g^{S}+g^{I}+Ng^{ES}},$$

(27) $$f_{G\text{d}}(g_{i}^{E})=\frac{g^{ES}g_{i}^{E}(E_{E}-E_{L})}{\left(g^{D}+g_{i}^{E})(g^{S}+Ng^{ES}\right)}$$

(28) $$\kappa_{\text{G}}=\frac{1}{E_{L}-E_{I}}.$$

Thus, the general multiplicative form of the shunting effect remains the same as in the on-path configuration of our initial model (Equation 11), but with the difference that now multiple excitatory inputs are first added together before being multiplied by the inhibitory component. Note that if more than one inhibitory input is considered (all similarly targeting the peri-somatic region), all contributions can be simply added up. That is *g*^I^ in Equation (25) can then be replaced by a sum of all inhibitory inputs, ∑_*j*_*g*^I^_*j*_.

### 2.3. Network dynamics with global shunting inhibition

In the above we have developed simple models describing the sub-threshold dynamics of point-neurons while integrating non-linear dendritic processing. These simplified single-compartment models are valuable to analyze the effects of shunting inhibition on the dynamics of large-scale networks.

**Figure 5:**
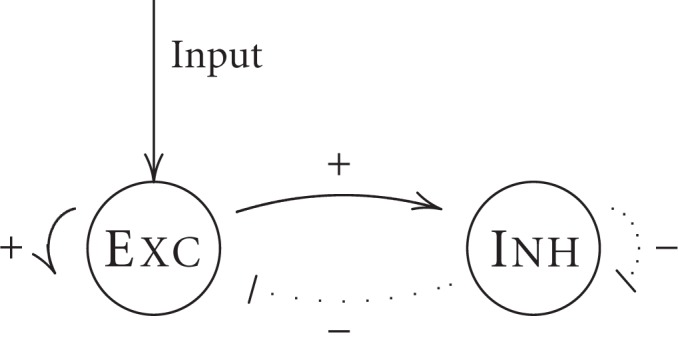
**A network model for generating persistent activity.** Neurons in excitatory (Exc.) and inhibitory (Inh.) groups are mutually connected in random and sparse fashion. Inhibitory effect are governed by a (peri-somatic) global shunting inhibition.

In the following, we will show by using our simplified neuron model that shunting inhibition might lead to persistent activity in spiking neuron networks as has been suggested previously for a different type of dendritic non-linearity (Abbott, 1991b). Our network structure of mutually interconnected groups of inhibitory and excitatory neurons (as illustrated in Figure 5) follows a common excitation-inhibition-type network layout (Wilson and Cowan, 1972; Brunel, 2000).

#### 2.3.1. Persistent activity by shunting inhibition

**Figure 6:**
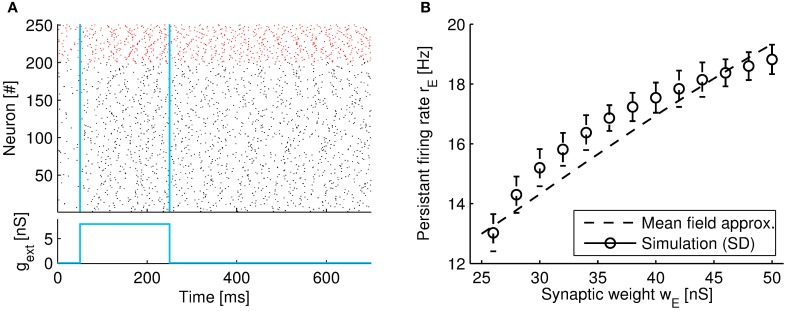
**Network showing persistent activity by shunting inhibition. (A)** Raster plot of the spiking activity of a selection of 250 neurons from the network. The *y*-axis plots the neuron index, where neurons 1–200 are excitatory (black color), and 201–250 are inhibitory (red color). After removal of the input (at *t* = 250 ms), the network retains persistent firing. The two vertical lines denote the onset and offset time of the external input, respectively (see margin plot below). The firing rates during persistent activity are *r*_*E*_ = 12.6 Hz, and *r*_*I*_ = 24.8 Hz (obtained by averaging the neural population over the time interval 400–500 ms). The synaptic weights are set to *w*_*E*_ = 24 nS and *w*_I_ = 2 nS. **(B)** The population-averaged firing rates of excitatory neurons vs. the excitatory connection strength *w*_*E*_. Simulation results averaged over 10 trials (circles; error bars indicate standard deviations) and the mean-field approximation (dashed line; Equation 64). Single neuron parameter as in Table 1. Other parameters: *N*^*E*^ = 2000, *N*^*I*^ = 500, *p* = 0.1, *C*^*E*^_S_ = 740 pF, *C*^I^_S_ = 370 pF, τ_E_ = 100 ms, τ_I_ = 10 ms, μ_*E*_ = 3.2, μ_I_ = 6.4, β = 17.5, *a* = 0.002, *b* = 0.175, *c* = −0.113, and *d* = −0.6218.

In detail, we built a network model of mutually connected *N*^*E*^ excitatory and *N*^*I*^ inhibitory neurons, with *N*^*E*^ = 4*N*^*I*^. Each neuron is sparsely connected to others with a small probability *p* = 0.1. For simplicity, the connection strengths between neurons are constants, with *w*_*E*_ and *w*_*I*_ denoting the excitatory and inhibitory strengths, respectively. We assume that each neuron in the circuit has *pN*^*E*^ branches, one for each excitatory input. All inhibitory inputs target the per-somatic region. In our model, both, excitatory and inhibitory neurons, are governed by non-linear dendritic processing and thus include shunting inhibition. Transfer conductances are assumed identical for each branch (for detailed parameters settings, see Figure 6).

According to Equation (25), the dynamics of the *i*th neuron in the network is

(29) $$\tau_{S}^{T}\frac{dv_{i}^{T}}{dt}=-(v_{i}^{T}-E_{L})+J_{i}^{T}+\sigma \xi_{i},$$

(30) $$\begin{array}{l} J_{i}^{T}=\sum_{j=1}^{pN^{E}}f_{G\text{d}}(g_{ij}^{E}+g^{\text{ext}})+f_{G\text{p}}\left(\sum_{j=1}^{pN^{I}}g_{ij}^{I}\right)\\ \;+\kappa_{\text{G}}f_{G\text{p}}\left(\sum_{j=1}^{pN^{I}}g_{ij}^{I}\right)\sum_{j=1}^{pN^{E}}f_{G\text{d}}(g_{ij}^{E}+g^{\text{ext}}) \end{array}$$

Here, the superscript *T* can either be *T* = *E* or *I* and denoted the type of the *i*th neuron. *J*^*T*^_*i*_ represents the total synaptic input received by the neuron, which consists of excitatory, inhibitory and shunting components. τ^*T*^_S_, *T* = *E* or *I*, are the somatic time constants of excitatory or inhibitory neurons, receptively, depending on the corresponding capacitance; we set *C*^*E*^_S_= 740 pF and *C*^*I*^_S_ = 370 pF. To model background noise, we include a Gaussian noise term of zero mean and variance σ^2^. *g*^ext^ refers to a small external input.

We further assume that a neuron fires a spike if its membrane potential reaches a threshold of *E*_thres_ = −50 mV. After a spike the membrane potential instantaneously is reset to *E*_reset_ = −70 mV. The dynamics of the excitatory and inhibitory synaptic conductance are given by Equations (7) and (8), respectively. We adjust the time constant of excitatory conductance to τ_E_ = 100 ms to account for the prominent role of slow NMDA conductances in the generation of persistent activity (Wang, 2002; Wong and Wang, 2006). Inhibitory conductance time scale is set to τ_I_ = 10 ms.

When parameters are chosen properly, we found that the network can retain sustained activity at a low firing rate even after an external stimulus is removed. An example of the network activity during persistent activity is shown in Figure 6A. Note that after a brief input (in the range 50–250 ms) the population firing rate remains high and does not return to the spontaneous activity level (which is induced by the noise term in the region 0–50 ms). Remarkably, the average population firing rate of the network does not reach very high firing rate but instead continuous to fire with a relatively modest rate of around 15 Hz for our parameter setting. An increase in the excitatory synaptic weight *w*_*E*_ causes the firing level to gradually increase (from 13 to 18 Hz in our example, see Figure 6B).

#### 2.3.2. Theoretical analysis of the persistent activity induced by global shunting inhibition

An intuitive picture for the underlying mechanism of the persistent activity induced by non-linear dendritic processing is as follows. When the firing rates of excitatory neurons are small, the self-excitation of excitatory neurons dominates and the firing rates of all neurons increase; however, with the excitation increasing further, the effect of shunting inhibition starts to grow dramatically in a non-linear manner due to its multiplicative dependence on the excitatory current. In case of persistent activity these two opposite effects are approximately balanced, so that neurons fire persistently at relatively low values without the need of external inputs.

To further elucidate these ideas, we carry out mean-field approximation by considering the average synaptic inputs to the excitatory and inhibitory populations, and approximate the network dynamics. The mean-field approach has previously been used to analyze the dynamical processes of neural networks with integrate-and-fire neurons (Amit and Brunel, 1997a,b; Brunel and Wang, 2001; Renart et al., 2003; Wong and Wang, 2006). Basically, as the network is composed of identical neurons, the synaptic input to each neuron in the network can be treated as a Gaussian random process. Thus we can use a single variable to represent population firing rates; let *r*_*E*_ and *r*_*I*_ denote the firing rates of excitatory and inhibitory neural populations, respectively. The population firing rates depend on synaptic currents, which in turn depends on firing rates. Thus, the population firing rate of a steady state can be determined by assuming self-consistency. In the analysis, we only consider the mean synaptic inputs to a neuron and neglect the input variance as it is of no importance in our setting (Wong and Wang, 2006).

From Equations (7) and (8) we find the dynamics of the excitatory and inhibitory synaptic conductances in the rate limit as

(31) $$\tau_{E}\frac{d{\tilde{g}}_{E}}{dt}=-{\tilde{g}}_{E}+w_{E}\tau_{E}r_{E}$$

(32) $$\tau_{I}\frac{d{\tilde{g}}_{I}}{dt}=-{\tilde{g}}_{I}+w_{I}\tau_{I}r_{I}.$$

If one then approximates the non-linear spiking mechanism by a threshold linear function (see e.g., Mongillo et al., 2008), *r*^*T*^ = μ^*T*^[*J*^*T*^ − β]_+_, with either *T* = *E* or *T* = *I*, we find from Equation (30)

(33) $$r_{E}=\mu_{E}{\left[J^{E}-\beta \right]}_{+},$$

(34) $$r_{I}=\frac{\mu_{I}}{\mu_{E}}r_{E}.$$

If one now uses linear functions to approximate *f*_*G*d_ and *f*_*G*p_ in the range of their average inputs according to Equation (30) one can straightforwardly solve for the population firing rate (see section 4.3.1). In our simulations, the mean-field approximation matched the simulation quite well (see Figure 6B, dashed line).

Finally, the stability of the fixed-point of the population firing rate can be analyzed (see section 4.3.2). It turns out that both eigenvalues are negative in our parameter settings, indicating stability. Moreover, the shunting strength κ_G_ has an stabilizing effect: with increasing κ_G_, eigenvalues will be decreased further. In summary, we confirm that our form of global shunting inhibition can cause spiking network models to exhibit persistent firing activity in a low rate regime.

## 3. Discussion

In the present study, we have presented a new derivation of a “dendritic-integrate-and-fire” single compartment neuron model. We show that the model captures well the non-linear integration of excitatory and inhibitory inputs at the soma with an arithmetic rule, where the shunting effect is expressed as a product between the contributions of excitatory and inhibitory inputs, as was suggested by recent experimental finding (Hao et al., 2009). In contrast to attempts relying on phenomenological modeling (Zhou et al., 2013), we start the derivation from a biophysical description of a three-compartment model, which allows for a biophysical interpretation of the equations. We find that the dependence of the shunting strength on the distance of the synaptic locations is well captured with the three-compartment model. In particular, in on-path configuration, the shunting strength decreases with the distance of the inhibitory site from the soma and stays constant with the distance of the excitatory site to the soma. These properties are found in experiments (Hao et al., 2009), but are not well captured by a simple two-port analysis, where the derived shunting strength is still dependent on a term related to the transfer conductance between excitatory site and the soma (Zhou et al., 2013). The reason for this result of a two-port analysis is that currents from both synaptic inputs are assumed to reach the soma. However, the real situation of the on-path configuration as captured by our three-compartment model is that currents from the distant excitatory compartment have to pass through the inhibitory compartment on its way. Thus there is no direct connection from excitatory site to soma as assumed by the simple two-port analysis as derived by Zhou et al. (2013).

In our derivation of the multiplicative rule of dendritic integration, we made two main assumptions: (1) the transfer conductances between the dendritic compartments in the antidromic direction are negligible small and (2) dendritic computations are fast in respect to the soma. The first assumption is only valid if the inhibitory and excitatory compartments are well separated on the dendritic tree. Thus the multiplicative rule refers only to the limit of well separated sites. Note that in the simulation (Figure 3B), the shunting strength is not constant for nearby input sites and the multiplicative rule is not applicable. Moreover, because of the depolarization for excitatory inputs, active channels increase the antidromic transfer to the inhibitory site in out-of-path configuration (which we modeled with the parameter α). Thus in comparison to on-path configuration, assumption (1) is less valid in out-of-path configuration (as illustrated in Figure 3A). Thus we conclude that the multiplicative rule can be mainly applied in the on-path configuration for well separated input sites.

Second, we further assumed that time scales of dendritic and somatic processing are separated, which is a good approximation because the large area of the soma in comparison to dendritic compartments. Whether the leak conductances per area of the somatic and dendritic compartments are on the same order or somewhat higher in the dendrite [as suggested experimentally (Golding et al., 2005; Omori et al., 2006, 2009)] is of no consequence to our analysis because the much larger membrane area of the soma will still ensure that the total leak is larger than in dendritic compartments. For our parameter setting, the time constant for the somatic membrane voltage is about 10 times slower than that of the dendritic compartments. Thus one can indeed assume that dendritic compartments instantly relax to steady state. Note that our assumption does not mean that the dynamics of synaptic conductances has to be fast. The dynamics of the synaptic input conductances were in fact not approximated and are still governed by their original form Equations (7) and (8). Thus our model can accommodate both, fast and slow ion-channels, such as AMPA and NMDA, respectively.

In a further analysis, we found that the arithmetic rule does much better apply to the on-path configuration than to the out-of-path configuration. In fact, in the latter case the shunting strength itself is dependent on the size of the inhibitory input and thus the arithmetic rule is only partly valid. This is admitted, but not further analyzed, also in the experimental study (Hao et al., 2009) where the authors state that the rule is valid only up to a certain range of input conductances.

Since our general aim was to derive a simple neuron model based on biophysical properties to be used for investigating the effects of shunting inhibition on network dynamics, we included active properties only as a multiplicative factor for certain transfer conductances to arrive at simple models. To model additional effects of active channels on shunting inhibition the voltage dependent dynamics of conductances have to be incorporated has been attempted in a recent study (Jadi et al., 2012) suggesting that information processing capabilities of dendritic integration might be even richer than thought previously for passive dendrites (Vu and Krasne, 1992).

### 3.1. Persistent activity

Our main aim of this study was to derive a simple model for analysis of network effects of non-linear dendritic processing. As an example of this approach, we investigated an simple network consisting of mutual connected groups of inhibitory and excitatory neurons and show that global shunting inhibition can naturally induce persistent activity.

Neuronal persistent firing has been widely observed in neural systems and is believed to play important roles in cognitive functions. For instance, persistent activity might hold information of a memory trace thereby storing information about recent inputs (Wang, 2001; Machens et al., 2005). It has been observed that during persistent activity, neurons fire irregularly at low rates, typically less than 100 Hz (Gold and Shadlen, 2001). From a dynamical systems point of view, to maintain self-sustained activity in a network, it needs, on one hand, strong excitatory interactions between neurons to retain the excitation, and, on the other hand, inhibitory interactions to avoid the divergence of neuronal responses. In a firing-rate based network model, one often assumes a sigmoidal input-output (IO) function to avoid the explosion of the network activity (e.g., Hopfield, 1984). In a network model of spiking neurons, the saturation of synaptic current mediated by NMDA receptor is often assumed to account for bounded neuronal responses (Wang, 2002). Although these models are capable of sustained activity, it seems energetically unlikely that the nervous system maintains activity in a regime where the saturation of neural properties is essential. Thus it is probably that other non-linear mechanisms ensure that run-away excitation does not occur.

In an earlier study, it was shown that a non-linear form of dendritic processing is indeed enough to generate persistent firing (Abbott, 1991b). Similar to our findings, the author accounted for non-linear dendritic processing in a network model and analyzed the resulting dynamics with a mean-field approach, concluding that persistent activity can occur in the low firing regime. The difference to our analysis is that the author starts from an even more biophysical detailed view (from the full cable equation) resulting in more detailed equations. The derivation thus does not yield the intuitive picture of a multiplicative rule weighted with a shunting strength parameter κ, as suggested experimentally. We here focused on the biophysically meaning of this rule and thus provided a different derivation starting from a three-compartment model and investigated its validity for different input configurations. The advantage of this rule [in contrast to equations suggested by Abbott (1991b)] is that two different non-linear effects of dendritic processing are clearly separated: First, the non-linear somatic response functions (see Figure 2), which comprise non-linear effects related to the leaky signal transduction of dendrites. Second, the multiplicative term of excitatory and inhibitory currents describing the shunting inhibition. By explicit weighting of the multiplicative term by a shunting strength κ both non-linearities can be individually analyzed.

We show that the multiplicative non-linearity of the shunting inhibition is needed to generate sustained activity without saturation. Note that the persistent activity found in our network does not rely on the non-linear form of the functions relating the input conductances to the somatic voltage change Figure 2, because further analysis shows that this saturation occurs at a firing rate much larger than 100 Hz and hence is not relevant (not shown).

In our formulation we used a factor α to effectively incorporate active channels. For global shunting, α mainly regulates the slope of the inhibitory somatic response equation (Equation 26) through the transfer conductance *g*^ES^, while the total excitatory response (summed over all *N* dendritic branches) is approximately independent of *g*^ES^ (see Equations 27 and 25). Although synaptic weights may need to be adjusted for a given persistent firing rate when α is varied, the qualitative picture of the network dynamics does not change.

Besides generating a sustained population activity of low firing rate, activity levels could also gradually adjusted by changing excitatory synaptic weights. Note that this graded change of activity level is not directly related to the “graded persistent activity” as found e.g., in the entorhinal cortex (Egorov et al., 2002). In the latter case, persistent activity level is changed by the amount of input given during a brief period. The underlying mechanism remains largely unknown (Brody et al., 2003). In contrast, in our case firing rate levels are adjusted by changing the recurrent synaptic weights.

This property of gradual adjustment of firing rates by varying the excitatory weight is induced by the strong multiplicative non-linearity during shunting. Note that the inhibitory effect mediated through shunting inhibition is multiplicative in both the excitatory and inhibitory rates. This square dependence induces a strong and robust change in the activity level when the synaptic weight is varied. On the other hand, if persistent activity is induced by a saturation of the firing rate IO-function, a change of the synaptic weight will have very little effect because the IO-function will be flat in the saturation limit.

In summary, our results suggest that shunting inhibition might be an important underlying mechanism in the dynamics of neural networks.

## 4. Materials and methods

### 4.1. The simplified neuron model

We consider first there is only excitatory synaptic input. By setting *g*^I^ = 0 in Equation (5), we have

(35) $$v^{I}-E_{L}=\frac{g^{SI}(v^{S}-E_{L})+g^{EI}(v^{E}-E_{L})}{g^{D}+g^{SI}+g^{EI}}.$$

Substituting Equation (6) into (35), we obtain

(36) $$\begin{array}{l} v^{I}-E_{L}=\frac{g^{SI}(v^{S}-E_{L})}{g^{D}+g^{SI}+g^{EI}}+\frac{g^{EI}}{g^{D}+g^{SI}+g^{EI}}\\ \;\times \frac{g^{IE}(v^{I}-E_{L})+g^{E}(E_{E}-E_{L})}{g^{D}+g^{E}+g^{IE}}. \end{array}$$

Re-organizing the above equation, we get

(37) $$\begin{array}{l} \text{​​​​​​​​​​​​​​}\left[1-\frac{g^{IE}g^{EI}}{\left(g^{D}+g^{SI}+g^{EI})(g^{D}+g^{E}+g^{IE}\right)}\right](v^{I}-E_{L})\\ \text{​​​​​​​​​​​​​​ }=\frac{g^{SI}(v^{S}-E_{L})}{g^{D}+g^{SI}+g^{EI}}+\frac{g^{EI}g^{E}(E_{E}-E_{L})}{\left(g^{D}+g^{SI}+g^{EI})(g^{D}+g^{E}+g^{IE}\right)}. \end{array}$$

Assuming *g*^IE^*g*^EI^ « (*g*^D^)^2^, we get

(38) $$\begin{array}{l} v^{I}-E_{L}\approx \frac{g^{SI}(v^{S}-E_{L})}{g^{D}+g^{SI}+g^{EI}}\\ \;+\frac{g^{EI}g^{E}(E_{E}-E_{L})}{\left(g^{D}+g^{SI}+g^{EI})(g^{D}+g^{E}+g^{IE}\right)}. \end{array}$$

Substituting Equation (38) into (4), we obtain

(39) $$\tau_{S}\frac{dv^{S}}{dt}=-(v^{S}-E_{L})+f_{\text{d}}(g^{E}),$$

where *f*_d_(*g*^E^) is given by Equation (12).

Similarly, we can calculate the case when there is only an inhibitory synaptic input by setting *g*^E^ = 0, and the result is

(40) $$\tau_{S}\frac{dv^{S}}{dt}=-(v^{S}-E_{L})+f_{\text{p}}(g^{I}),$$

where *f*_p_(*g*^I^) is given by Equation (13).

Finally, by combing the above results, we obtain the dynamics of the somatic potential when both excitatory and inhibitory synaptic inputs are applied (see Equation 11)

(41) $$\tau_{S}\frac{dv^{S}}{dt}=-(v^{S}-E_{L})+f_{\text{d}}(g^{E})+f_{\text{p}}(g^{I})+\kappa f_{\text{d}}(g^{E})f_{\text{p}}(g^{I}),$$

where κ is given by Equation (14).

The key algebraic insight in the derivation of the multiplicative rule for the integration of the synaptic input conductances, Equation (41), is the following. We exemplify the key step in a simplified system. Note first that dendritic processing in the inhibitory compartment is linearly transmitted into the somatic compartment, given by Equation (4). We thus can focus on the steady state equation of the inhibitory compartment, as given by Equation (9) and set for now *g*^SI^ = 0. If we assume that the transfer conductance back to the excitatory compartment is negligible [that is *g*^IE^*g*^EI^ « (*g*^D^)^2^ as above], the excitatory voltage is not dependent on the inhibitory voltage. Thus it is

(42) $$v^{-I}-E_{L}\approx \frac{g^{EI}(v^{-E}-E_{L})+g^{I}(E_{I}-E_{L})}{g^{D}+g^{SI}+g^{EI}+g^{I}}$$

(43) $$=\frac{h(g^{E})+\beta g^{I}}{\gamma +g^{I}}$$

where we set *h*(*g*^E^) ≡ *g*^EI^(*v*^ − *E*^ − E_L_), γ ≡ *g*^D^ + *g*^SI^ + *g*^EI^, and β ≡ *E*_I_ − *E*_L_, for the time being. Crucially, inhibition and excitation terms in Equation (43) are mixed due to the *g*^I^ in the denominator. The key insight is that the term (Equation 43) can be expressed as a multiplicative rule separating the effects of *g*^I^ and *g*^E^:

(44) $$\frac{h(g^{E})+\beta g^{I}}{\gamma +g^{I}}=\frac{\beta g^{I}}{\gamma +g^{I}}+\frac{h(g^{E})}{\gamma +g^{I}}$$

(45) $$=\frac{\beta g^{I}}{\gamma +g^{I}}+\frac{h(g^{E})}{\gamma +g^{I}}-\frac{h(g^{E})}{\gamma }+\frac{h(g^{E})}{\gamma }$$

(46) $$=\frac{\beta g^{I}}{\gamma +g^{I}}+\frac{h(g^{E})}{\gamma }+h(g^{E})\left(\frac{1}{\gamma +g^{I}}-\frac{1}{\gamma }\right)$$

(47) $$=\frac{\beta g^{I}}{\gamma +g^{I}}+\frac{h(g^{E})}{\gamma }-\frac{h(g^{E})g^{I}}{\gamma (\gamma +g^{I})}$$

(48) $$=\frac{\beta g^{I}}{\gamma +g^{I}}+\frac{h(g^{E})}{\gamma }-\frac{1}{\beta }\frac{\beta g^{I}}{\gamma +g^{I}}\frac{h(g^{E})}{\gamma }$$

(49) $$\equiv f_{1}(g^{I})+f_{2}(g^{E})-\frac{1}{\beta }f_{1}(g^{I})f_{2}(g^{E})$$

which has the desired multiplicative form with *f*_1_(*g*^I^) ≡ *βg*^I^/(γ + *g*^I^) and *f*_2_(*g*^E^) ≡ *h*(*g*^E^)/γ. The derivation of Equation (41) was done analogously albeit starting from the full model (including the somatic compartment).

### 4.2. The dynamics of a neuron having multiple dendrites

#### 4.2.1. Single synapses on individual dendritic branches

We first consider that single synaptic inputs are distributed on parallel dendritic branches, i.e., each branch receives only one type of synaptic input (see Figure 4A). In this case, the dynamics of the neural compartments can be written as

(50) $$C_{S}\frac{dv^{S}}{dt}=-g^{S}(v^{S}-E_{L})-\sum_{i}g^{TS}(v^{S}-v_{i}^{T})$$

(51) $$\begin{array}{l} C_{{}^{D}}\frac{dv_{i}^{T}}{dt}=-g^{D}(v_{i}^{T}-E_{L})-g^{ST}(v_{i}^{T}-v^{S})-g_{i}^{T}(v_{i}^{T}-E_{i}^{T}),\\ \;i=1,2,\ldots N. \end{array}$$

Here, we use *T* = *E* or *I* to denote the type of an input synapse. *v*^T^_*i*_ represent the potential at the location of the *T*-type input on the *i*th dendrite. *g*^ST^ is the transfer conductance from the soma to an input site, which we assume equal for different dendritic branches. *g*^TS^ is defined analogously.

Given that the dynamics of the voltage of the synaptic compartments, *v*^*T*^_*i*_, happens on much faster time scale than the somatic voltage *v*^S^, we can assume that they instantaneously relax to the steady state. By thus setting the left-hand side of Equation (51) to zero, we get

(52) $$v_{i}^{T}-E_{L}=\frac{g^{ST}(v^{S}-E_{L})+g_{i}^{T}(E_{i}^{T}-E_{L})}{g^{D}+g^{ST}+g_{i}^{T}}.$$

Substituting Equation (52) into (50) and assuming that *g*^TS^*g*^ST^ « (*g*^S^)^2^ leads to the model as stated in the main text (Equation 19).

#### 4.2.2. On-path configuration on each dendritic branch

We now consider the second configuration in which each dendrite branch comprises of a pair of excitatory and inhibitory inputs in on-path arrangement, that is the inhibitory synapse is located proximal and the excitatory synapse distal (in respect to the soma; see Figure 4B). The voltage dynamics of all compartments is then given by

(53) $$C_{S}\frac{dv^{S}}{dt}=-g^{S}(v^{S}-E_{L})-\sum_{i}g^{IS}(v^{S}-v_{i}^{I})$$

(54) $$\begin{array}{l} C_{{}^{D}}\frac{dv_{i}^{I}}{dt}=-g^{D}(v_{i}^{I}-E_{L})-g^{SI}(v_{i}^{I}-v^{S})\\ \;-g^{EI}(v_{i}^{I}-v_{i}^{E})-g_{i}^{I}(v_{i}^{I}-E_{I}) \end{array}$$

(55) $$\begin{array}{l} C_{{}^{D}}\frac{dv_{i}^{E}}{dt}=-g^{D}(v_{i}^{E}-E_{L})-g^{IE}(v_{i}^{E}-v_{i}^{I})-g_{i}^{E}(v_{i}^{E}-E_{E}),\\ \;i=1,2,\ldots N \end{array}$$

where *v*^E^_*i*_ and *v*^I^_*i*_ are the membrane potentials at the locations E and I, and *g*^E^_*i*_ and *g*^I^_*i*_ are the excitatory and inhibitory synaptic conductances of the *i*th dendritic branch, respectively.

Consider again that the voltages at the dendritic locations, *v*^E^_*i*_ and *v*^I^_*i*_, can be regarded as fast variables, Equations (54) and (55) can be solved for the stationary state of *v*^E^_*i*_ and *v*^I^_*i*_, respectively. Substituting the results into Equation (53), and again assuming that the product of the transfer conductances are much smaller than the leaks, *g*^SI^*g*^IS^ « (*g*^S^)^2^ and *g*^IE^*g*^EI^ « (*g*^D^)^2^, we find the final form of the model as given in the main text (Equation 21).

#### 4.2.3. Global shunting

In the third configuration, we consider that an inhibitory input targets the soma directly and excitatory inputs are distributed on parallel dendritic branches (see Figure 4C). The dynamics of the compartments can be written as

(56) $$\begin{array}{l} C_{S}\frac{dv^{S}}{dt}=-g^{S}(v^{S}-E_{L})-g^{I}(v^{S}-E_{I})\\ \;-\sum_{i}g^{ES}(v^{S}-v_{i}^{E}), \end{array}$$

(57) $$\begin{array}{l} C_{{}^{D}}\frac{dv_{i}^{E}}{dt}=-g^{D}(v_{i}^{E}-E_{L})-g^{SE}(v_{i}^{E}-v^{S})\\ \;-g_{i}^{E}(v_{i}^{E}-E_{E}),\;i=1,2,\ldots N. \end{array}$$

Note that we do not need to explicitly model an inhibitory compartment here because the inhibitory input directly affects the somatic potential. Thus the somatic time constant, given by τ_S_ = *C*_S_/(*g*^S^ + *g*^IS^) when explicitly modeling the inhibitory compartment, is now approximated by τ_S_ ≈ *C*_S_/*g*^S^ since the transfer conductance from inhibitory compartment to the soma *g*^IS^ is neglected. Following analogous assumptions as above we get the Equation (25) in the main text.

### 4.3. Persistent activity in a network of global shunting gates

#### 4.3.1. Fixed point of population firing rates

We first approximate the functions *f*_*G*d_ and *f*_*G*p_ in the range of their average inputs, that is ${\tilde{g}}_{E}$ and $N^{I}p{\tilde{g}}_{I}$ [compare to Equation (30)]. This yields $f_{G\text{d}}({\tilde{g}}_{E})\approx a{\tilde{g}}_{E}+b$ and $f_{G\text{p}}(N^{I}p{\tilde{g}}_{I})\approx cN^{I}p{\tilde{g}}_{I}+d$, where *a* > 0, *b* ≥ 0, *c* < 0, *d* ≤ 0 are constants (see Figure 6 for the numerical values). Now we find for the inputs:

(58) $$\begin{array}{l} J^{T}=N^{E}p(a{\tilde{g}}_{E}+b)+cN^{I}p{\tilde{g}}_{I}+d\\ \;+\kappa_{\text{G}}N^{E}p(a{\tilde{g}}_{E}+b)(cN^{I}p{\tilde{g}}_{I}+d) \end{array}$$

Since the conductances and population rates have to match during a steady state (self-consistency), we can in the following calculate the fixed point of the population rates, ${\bar{r}}_{E}$ and ${\bar{r}}_{I}$. Using Equation (58) together with the steady states of Equations (31) and (32), we find first for the inputs

(59) $$J^{T}=A_{1}{\bar{r}}_{E}+A_{2}{\bar{r}}_{I}+A_{3}{\bar{r}}_{E}{\bar{r}}_{I}+A_{4},$$

with the shortcuts

(60) $$A_{1}=aw_{E}\tau_{E}pN^{E}(1+d\kappa_{\text{G}})$$

(61) $$A_{2}=cw_{I}\tau_{I}pN^{I}(1+b\kappa_{\text{G}}pN^{E})$$

(62) $$A_{3}=\kappa_{\text{G}}\;aw_{E}\tau_{E}pN^{E}\;cw_{I}\tau_{I}pN^{I}$$

(63) $$A_{4}=bpN^{E}(1+d\kappa_{\text{G}})+d.$$

Combining Equation (59) with Equations (33) and (34) finally yields the fixed point of the population firing rates

(64) $${\bar{r}}_{E}=\frac{2B_{1}}{-B_{2}+\sqrt{B_{2}^{2}-4B_{1}B_{3}}},$$

(65) $${\bar{r}}_{I}=\frac{\mu_{I}}{\mu_{E}}{\bar{r}}_{E},$$

where *B*_1_ = μ_*E*_ *A*_4_ − μ_*E*_ β, *B*_2_ = μ_*E*_ *A*_1_ + μ_*I*_ *A*_2_ −1, and *B*_3_ = μ_*I*_ *A*_3_.

#### 4.3.2. Stability

Let us analyze the stability of the stationary states, Equations 64 and (65). We thus have to consider the following dynamical equations

(66) $$\tau_{E}\frac{d{\tilde{g}}_{E}}{dt}=-{\tilde{g}}_{E}+w_{E}\tau_{E}r_{E}=:G_{1}({\tilde{g}}_{E},{\tilde{g}}_{I})$$

(67) $$\tau_{I}\frac{d{\tilde{g}}_{I}}{dt}=-{\tilde{g}}_{I}+w_{I}\tau_{I}r_{I}=:G_{2}({\tilde{g}}_{E},{\tilde{g}}_{I})$$

The fixed points of the above equations are

(68) $${\bar{\tilde{g}}}_{E}={\bar{r}}_{E}w_{E}\tau_{E}$$

(69) $${\bar{\tilde{g}}}_{I}=\frac{{\bar{r}}_{E}w_{I}\tau_{I}\mu_{I}}{\mu_{E}}$$

The stability of the population activity is determined by the eigenvalues of the Jacobian matrix *J* at the fixed points. The Jacobian matrix is given by

(70) $$J={\left[\begin{array}{cc} \frac{\partial G_{1}}{\partial {\tilde{g}}_{E}} & \frac{\partial G_{1}}{\partial {\tilde{g}}_{I}}\\ \frac{\partial G_{2}}{\partial {\tilde{g}}_{E}} & \frac{\partial G_{2}}{\partial {\tilde{g}}_{I}} \end{array}\right]}_{\left({\bar{\tilde{g}}}_{E},{\bar{\tilde{g}}}_{I}\right)}$$

(71) $$=\left[\begin{array}{cc} -1+N^{E}pw_{E}\tau_{E}\mu_{E}a(1+\kappa_{\text{G}}d+\kappa_{\text{G}}cN^{I}p{\bar{\tilde{g}}}_{I}) & N^{I}pw^{E}\tau_{E}\mu_{E}c(1+\kappa_{\text{G}}N^{E}pb+\kappa_{\text{G}}N^{E}pa{\bar{\tilde{g}}}_{E})\\ N^{E}pw_{I}\tau_{I}\mu_{I}a(1+\kappa_{\text{G}}d+\kappa_{\text{G}}cN^{I}p{\bar{\tilde{g}}}_{I}) & -1+N^{I}pcw^{I}\tau_{I}\mu_{I}(1+\kappa_{\text{G}}N^{E}pb+\kappa_{\text{G}}N^{E}pa{\bar{\tilde{g}}}_{E}) \end{array}\right]$$

whose eigenvalues are calculated to be

(72) $$\lambda_{1}=-1,$$

(73) $$\begin{array}{l} \lambda_{2}=N^{E}pw_{E}\tau_{E}\mu_{E}a(1+\kappa_{\text{G}}d+\kappa_{\text{G}}N^{I}pc{\bar{\tilde{g}}}_{I})\\ \;+\;N^{I}pcw_{I}\tau_{I}\mu_{I}(1+\kappa_{\text{G}}N^{E}pb+\kappa_{\text{G}}N^{E}pa{\bar{\tilde{g}}}_{E})-1. \end{array}$$

Thus, when λ_2_ < 0 the solution is stable. Note that since only *c* < 0 and *d* ≤ 0 the increasing shunting strength κ_G_ will decrease λ_2_ and thus tend to stabilizing the system.

### Conflict of interest statement

The authors declare that the research was conducted in the absence of any commercial or financial relationships that could be construed as a potential conflict of interest.
